# Empowering Aging Minds: Advancing Health Literacy Among Older Adults Through Community-Based Education

**DOI:** 10.51894/001c.144139

**Published:** 2025-09-30

**Authors:** Renell Shisha

**Affiliations:** 1 College of Osteopathic Medicine Michigan State University

## 42

### BACKGROUND

Health literacy plays a crucial role in the well-being of older adults, shaping their ability to comprehend medical information, make informed decisions, and effectively manage their health. Limited health literacy is associated with medication errors, poor disease management, and adverse health outcomes. As the aging population grows, addressing health literacy is increasingly important.

This ongoing quality improvement (QI) initiative seeks to assess and enhance health literacy among older adults in the Metro-Detroit area through community-based participatory research (CBPR). By engaging with local communities, this proposal aims to share a methodology to establish a replicable framework for sustainable educational strategies that aid older adults in navigating their healthcare needs.

### OBJECTIVES

The goal of this QI project is to develop a model for assessing health literacy needs in older adults and implementing targeted educational interventions to enhance their understanding of health information. By establishing a structured framework, this project has the potential to be replicated and adapted across diverse settings, effectively supporting older populations.

### METHODS

Monthly seminars will be implemented at Senior Adult Life Centers in the Metro-Detroit area. Psychiatry residents, faculty, and medical students from Michigan State University will lead sessions on mental, physical, public and sexual health. The seminars will incorporate plain language, visual aids, and interactive activities to facilitate comprehension. Pre- and post-seminar surveys, featuring Likert scale and open-ended questions, will assess health literacy levels, confidence in health management, and understanding of key topics. Data analysis will use a cross-sectional analytical design to evaluate intervention effectiveness.

### EXPECTED RESULTS

Survey data are expected to demonstrate significant improvements in participants’ health literacy, particularly their confidence in managing health and understanding complex health concepts. Preliminary results are expected to indicate positive changes in participants’ ability to navigate healthcare systems, supporting the effectiveness of the educational interventions.

### CONCLUSION

By addressing health literacy gaps with targeted interventions, this project has the potential to improve health outcomes for older adults. If successful, this model, using a CBPR approach, could be replicated to guide future initiatives assessing long-term impacts on health behaviors and healthcare utilization, while adapting to meet the evolving needs of aging populations.

